# Pancreatic Cancer and Primary Sjögren's Syndrome: A Case Report

**DOI:** 10.1155/2021/9915881

**Published:** 2021-07-31

**Authors:** Daniela Oliveira, Vanessa Chaves, José Carlos Martins, Carlos Vaz, Miguel Bernardes, Jorge Almeida

**Affiliations:** ^1^Rheumatology Department, University Hospital Center of São João, Porto, Portugal; ^2^Center for Health Technology and Services Research (CINTESIS), Faculty of Medicine, University of Porto, Porto, Portugal; ^3^Internal Medicine Department, University Hospital Center of São João, Porto, Portugal; ^4^Department of Medicine of Faculty of Medicine, University of Porto, Porto, Portugal

## Abstract

Primary Sjögren syndrome (SS) is a chronic inflammatory systemic autoimmune disease with a high risk of malignancy development, namely, lymphoproliferative neoplasms. Few studies also reported a high risk of solid cancers; however, the coexistence of primary SS and pancreatic cancer has been rarely described. In this paper, we aim to describe a case of a 59-year-old woman who was an active smoker with sicca symptoms and symmetrical polyarthritis and was diagnosed with primary SS two years before the development of metastatic pancreatic adenocarcinoma. Despite institution of chemotherapy, the patient succumbed to the malignancy. Besides that, we explore the link between primary SS and solid cancers including the main predictors of malignancy and the role of primary SS as a paraneoplastic syndrome. Patients with primary SS should be closely monitored for malignancy, not only for hematological cancer, but also for solid tumors. Further research is necessary to understand which are the predictors of cancer proliferation in primary SS patients.

## 1. Introduction

Sjögren syndrome (SS), firstly described in 1933, is a chronic inflammatory systemic autoimmune disease that mainly affects middle-aged women. SS can be primary, when it occurs in a patient who does not have another underlying rheumatic disease, or secondary when there is an associated connective tissue disease. Patients with SS experience xerostomia and xerophthalmia that results from lymphocytic cells infiltrate and autoimmune destruction of the exocrine glands, such as salivary, lachrymal, and parotid glands. However, any exocrine gland can be involved in SS and the hypothesis of a pancreatic exocrine dysfunction has been explored.

Previous research explored a link between rheumatic diseases, autoimmunity, and cancer, concluding that chronic autoimmune inflammation is associated with cancer development. In primary SS, the most common malignant neoplasms are hematological, namely, non-Hodgkin B-cell lymphomas [1, 2]. Moreover, in patients with SS, several studies reported a higher risk for solid cancers, such as oral cavity, nasopharyngeal, thyroid, and stomach tumors [1, 3–8]. Despite the increased risk of solid cancers and pancreatic exocrine dysfunction in these patients, the coexistence of primary SS and pancreatic cancer was rarely reported. Yet, it is known that some rheumatic diseases such as inflammatory myopathies, vasculitis, and scleroderma-like syndromes may be associated with or precede the clinical manifestations of a variety of solid cancers and may represent a paraneoplastic syndrome [9]. However, to the best of our knowledge, there are only a few case reports supporting the hypothesis that SS may represent a paraneoplastic syndrome [10, 11].

Therefore, it is imperative to collect, describe, and discuss more clinical case reports to support the link between primary SS and solid cancer in order to inform non-retrospective studies in the future that adequately explore this association over time. Thus, this study aimed to describe a case of a woman previously diagnosed with primary SS that developed a metastatic pancreatic adenocarcinoma and to discuss the link between primary SS and solid cancers.

## 2. Case Presentation

A Caucasian 59-year-old woman, current smoker (10 pack-years), presented with symptomatic dry eye and mouth and inflammatory arthralgia. The patient had no known personal or family history of rheumatic disease. Physical examination at rheumatology consultation revealed bilateral conjunctival congestion and symmetrical polyarthritis affecting proximal interphalangeal and metacarpophalangeal joints. Although sicca symptoms are commonly present in smokers [12], this patient also had polyarthritis on physical examination, and for this reason, the investigation for SS was performed. Serum levels of C-reactive protein (CRP), erythrocyte sedimentation rate (ESR), C3 and C4, IgA, IgM, and proteinogram were normal. IgG1, IgG3, and IG4 were normal; IgG2 was equal to 787 (normal range, 171 to 632 mg/dL). Rheumatoid factor (RF), antinuclear antibodies, and anti-SSA/B antibodies were negative. Hands conventional radiography revealed no erosions. Additional investigation for SS showed decreased parotid gland function on salivary glands scintigraphy, and ophthalmologic examination with Schirmer's test and tear break-up time confirmed severe dry eye. Lower labial mucosa biopsy revealed focal chronic sialadenitis characterized by intense lymphocytic inflammatory infiltrate (1 focus/4 mm^2^ of glandular tissue). These findings (Schirmer's test and focal labial mucosa biopsy) were consistent with the classification of primary SS according to the American College of Rheumatology/European League Against Rheumatism criteria (2016). The patient did not tolerate hydroxychloroquine and was treated with prednisolone 5 mg/daily and pilocarpine 5 mg four times a day. The patient was evaluated every 6 months, and her symptoms partially improved under these medications.

**Figure 1:**
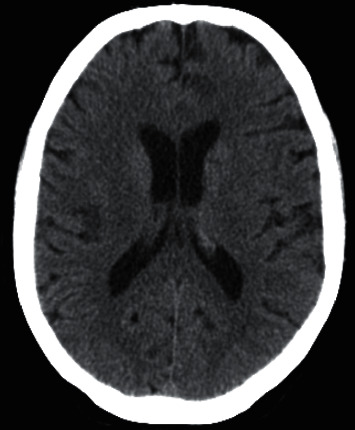
Ischemic stroke affecting multiple vascular territories.

About two years after the diagnosis of primary SS, the patient started complaining of epigastric pain, nausea, and severe low back pain. She also had asthenia and weight loss (15 kg in 5 months). After a long-haul flight, she developed lower limb deep vein thrombosis and was hypocoagulated with dabigatran. Ten days later, the patient was admitted in the emergency department with an ischemic stroke in multiple vascular territories (Figure 1). Physical examination revealed a left lower facial paresis and dysarthria. From the stroke study, electrocardiogram revealed sinus rhythm, cervical and transcranial Doppler ultrasound, a total occlusion of the right middle cerebral artery, and transthoracic echocardiography, a redundant oval foramen membrane with a positive right-left shunt. Additional investigation showed normocytic anemia (Hb 10.4 g/dL), leukocytosis (18.000/*μ*L) with neutrophilia (90.3%/13.500/*μ*L), thrombocytosis (510.000/*μ*L), and elevated CRP (73 mg/L) and ESR (62 mm in the first hour). Over time, a progressive elevation of liver cholestasis enzymes occurred: gamma-glutamyl transferase 534 (normal range, 7 to 32 U/L) and alkaline phosphatase 357 (normal range, 30 to 120 U/L), with no cytolysis. Pancreatic enzymes, renal function, proteinogram, and urinalyses were normal. Viral serologies were negative. Abdominal ultrasound and upper and lower gastrointestinal endoscopies revealed no pathological findings. Subsequent cervical, chest, abdomen, and pelvic computed tomography scan (TC CCAP) showed non-specific scattered pulmonary micronodules in lung lower lobes, liver parenchyma with several nodules, and retroperitoneal ganglion cluster in the hepatic hilum.

According to these findings, the diagnostic hypotheses postulated were neoplastic metastasis of a solid tumor or lymphoproliferative disease. In this regard, mammography, peripheral blood immunophenotyping, and bone marrow biopsy were performed which showed no evidence of a neoplastic disorder. Liver biopsy was subsequently performed, and pathologic analysis revealed an adenocarcinoma.

In accordance with the European Society for Medical Oncology and considering the diagnosis of an occult primary neoplasm with unfavorable histological subtype, the patient started an empirical chemotherapy protocol with carboplatin and paclitaxel. After the first cycle, treatment was suspended because the patient experienced a deterioration of her general clinical condition with progressive worsening of cytocholestasis parameters. The patient performed a new TC CCAP that revealed moderate ectasia of biliary tract and pancreatic duct, demonstrating one mass adjacent to the pancreatic head, which may indicate the epicenter of the neoplastic process. About one month after the first chemotherapy cycle, the patient died with a diagnosis of probable metastatic pancreatic adenocarcinoma.

## 3. Discussion

Primary SS is associated with an elevated risk of developing malignant neoplasms, with an overall incidence of all cancers being 2.6 times that of the general population [2]. This increased risk of cancer is due to systemic inflammation, loss of protective secretions, and persistent tissue damage and repair. The most common neoplasms reported are lymphoproliferative disorders, associated with a continued abnormal stimulation of B cells in the exocrine glands, affecting about 5% of patients [1, 4, 7, 13]. Retrospective studies confirmed that there is an increased incidence of non-Hodgkin B-cell lymphoma and multiple myeloma in these patients [1, 2]. Although solid cancers such as oral cavity tumors [1, 4, 6, 14], nasopharyngeal carcinoma [5, 15], thyroid cancer [3, 4, 6, 7, 15], thymoma [1, 16, 17], breast cancer [1, 7, 14, 18], lung cancer [6, 15], and stomach cancer [4, 8, 19] have been described, as presented in Table 1, most of these tumors were diagnosed during the first two years following the diagnosis of primary SS [3]. Nationwide population-based studies observed that for site-specific solid cancers, oropharynx, nasopharyngeal, lung, and thyroid cancers were highest in patients with SS [3, 6, 15]. However, the available data about an association between primary SS and solid cancers is controversial, and, in some studies, this association is not statistically significant [2, 12, 20, 21]. A recent meta-analysis (2019) concluded that primary SS is not associated with non-lymphoma malignancy risk [23]. The small sample size and a retrospective design of some previous studies have been pointed out as the main reasons for the lack of this association.

To our knowledge, the coexistence of primary SS and pancreatic cancer was described only in two recent studies, with a prevalence of pancreatic cancer equal to 0.8% [7] and a crude incidence of 10.0 (95% confidence interval 5.6–14.4) [6]. Nevertheless, there is a crucial concept of “autoimmune exocrinopathy” in which the pancreas is an exocrine gland that is functionally and histologically comparable to the salivary glands and pancreatic dysfunction is common in patients with primary SS. About 50% of thesepatients have an elevated level of at least one pancreatic enzyme, and an abnormal pancreatic ductal configuration was reported in 27% of them [24]. However, few studies have explored the clinical implications of pancreatic dysfunction. Patients with primary SS have an increased risk of acute pancreatitis, and the elevated IgG4 serum levels in these patients increased the prevalence of autoimmune chronic pancreatitis and cholangitis [25].

In this sense, we know from previous literature that there is an increased incidence of malignancies in patients with IgG4-related disease, especially of pancreatic cancer in IgG4-related pancreatitis. Furthermore, previous study provides evidence about Ro60/SSA targeted in SS, which can facilitate pancreatic cancer proliferation, migration, and invasion [26]. Conversely, the patient described in this case had normal levels of pancreatic enzymes, negative anti-SSA/B antibodies, and increased IgG2 levels (and not IgG4).So, further research is necessary to understand which are the others predictors of pancreatic dysfunction and cancer proliferation in primary SS patients.

### 3.1. Predictors of Cancer in Primary SS

Several independent predictors of hematological cancer development have been identified, namely, male gender, Raynaud phenomenon, palpable purpura, lymphadenopathy, parotid enlargement, vasculitis, splenomegaly, cryoglobulinemia, low C3 and C4 levels, anti-SSA/B antibodies, rheumatoid factor (RF) positivity, monoclonal gammopathy, disease duration, increased disease activity, and daily use of corticosteroids [1, 2, 4, 18, 27–31].

SS patients who develop lymphomas appear to be at increased risk of developing additional cancers. This potential association may be due to different reasons: defective DNA repair mechanisms, suppressed immunity, and cytotoxic treatments [2]. Although no variables were significantly associated with the development of solid cancer in primary SS patients [4], a recent study identified significant variables associated with overall malignancy, namely, thrombocytopenia and low C3 levels [19]. A more recent study characterized for the first time the SS/scleroderma autoantigen 1 (SSSCA1), an autoantigen overexpressed in SS and a potential marker of various solid cancers, such as colorectal, breast, cervical, lung, and prostate [32]. Further, a nationwide population-based study verified that the incidence of cancer among SS patients increased with age and male patients had overall higher risk than female patients [3]; another study verified that the ethnicity (non-white women) was the unique risk factor for the development of any solid cancer [4]. Further research is needed to explore the predictive factors of non-hematological cancers in primary SS in order to develop screening strategies able to timely diagnose and treat these patients.

### 3.2. Primary SS as a Paraneoplastic Syndrome

The case described a patient presented initially with polyarthritis and a primary SS diagnosis that preceded two years the diagnosis of an exocrine gland adenocarcinoma, and this fact can raise the hypothesis of primary SS as a paraneoplastic rheumatic syndrome. The most frequent malignancies associated with paraneoplastic rheumatic syndrome are hematological, although lung, breast, colon, ovarian, and stomach cancers have also been described. The pathogenesis seems to be complex and related with several hypotheses: neoplasia and paraneoplastic rheumatic syndrome having the same trigger, tumor cells producing inflammatory toxins, and a hypersensitivity reaction against intracellular antigens secreted by apoptotic cancer cells [9].

Seronegative symmetrical inflammatory polyarthritis involving the small joints of the hands and wrists and Raynaud phenomenon are common rheumatic manifestation of paraneoplastic syndromes. However, various other syndromes such as hypertrophic osteoarthropathy, inflammatory myopathies, vasculitis, lupus-like syndrome, scleroderma-like syndrome, and SS were described. In particular, the paraneoplastic meaning of primary SS remains poorly understood. One study which aimed to explore the spectrum of rheumatic diseases in breast cancer showed that 5.5% of women had SS [22]. To the best of our knowledge, there are only two case reports in the literature exploring SS as a paraneoplastic syndrome preceding lung and renal cancers [10, 11]. Thus, as exemplified in our case, a rapid onset of an unusual inflammatory polyarthritis in a patient older than 50 years with smoking habits, with partial response to low-dose corticosteroid, and with no specific biologic or radiographic features should lead to screening of an occult neoplasm [9].

## 4. Conclusion

In conclusion, solid cancers have been associated with primary SS; therefore, these patients should be closely monitored, not only for the enhanced risk of hematological cancer, but also for some types of these cancers. To the best of our knowledge, the presented report is the first one to extensively describe and discuss a case of a pancreatic cancer in a patient with primary SS. However, in this patient, the presence of smoking habits is also a risk factor for the development of lung cancer, and this fact constitutes a limitation in the description and discussion of this clinical case [33]. Some patients develop paraneoplastic rheumatic syndromes, and the possibility of an atypical rheumatic manifestation with an underlying neoplastic process should always be considered. Primary SS as a paraneoplastic syndrome remains poorly understood, and more studies are needed to explore this association.

## Figures and Tables

**Table 1:** Main original studies assessing solid cancer risk in primary SS.

Author, country, year	Study design	Number of patients with primary SS included	Type of malignancy associated
Insler and Shelin; USA; 1987 [17]	Case report	1	Thymoma
Janssens et al.; Belgium; 1987 [11]	Case report	1	Primary SS as paraneoplastic syndrome
Matsumoto et al; Japan; 1996 [16]	Case report	1	Thymoma
Theander et al.; Sweden; 2006 [20]	Retrospective cohort	507	Lymphoproliferative malignancy
Lazarus et al.; England; 2006 [2]	Retrospective cohort	112	Lymphoproliferative and multiple myeloma
Gadalla et al.; USA; 2009 [21]	Retrospective case -control	196^*∗*^	Breast cancer
Zhang et al.; China; 2010 [1]	Retrospective cohort	1320	Lymphoproliferative malignancy, multiple myeloma; thymoma; oral cavity and breast
Bartoloni et al.; Italy; 2012 [10]	Case report	1	Primary SS as paraneoplastic syndrome
Weng et al; Taiwan, 2012 [3]	Retrospective cohort	7852	Thyroid
Boussios et al.; Greece; 2014 [13]	Retrospective cohort	450	Lymphoproliferative malignancy
Lai et al.; Taiwan 2014 [5]	Case report	1	Nasopharyngeal
Yu et al.; Taiwan; 2016 [15]	Retrospective cohort	11998	Nasopharyngeal, thyroid, and lung
Brito Zéron et al.; Spain; 2017 [4]	Retrospective cohort	1300	Oral cavity, thyroid, and stomach
Brom et al.; Argentine; 2019 [14]	Retrospective cohort	157	Oral cavity and breast
Tarhan et al.; Turkey; 2019 [22]	Retrospective cohort	8^*∗∗*^	Breast
Findakly; USA; 2020 [8]	Case report	1	Gastric adenocarcinoma
Kang et al.; South Korea; 2020 [6]	Retrospective cohort	6359	Oropharynx, thyroid, liver, and lung (more common); pancreatic cancer
Mathews et al.; USA; 2020 [7]	Retrospective cohort	126	Lymphoproliferative malignancy, breast, and thyroid (more common); pancreatic cancer

^*∗*^Out of 84,778 breast cancer patients; ^*∗∗*^out of 128 breast patients.
